# Evaluating the long-term efficacy and effectiveness of Viscocanalostomy and combined phacoemulsification with Viscocanalostomy in the treatment of patients with uveitic glaucoma: 5-year follow up data

**DOI:** 10.1186/s12893-021-01176-5

**Published:** 2021-04-19

**Authors:** Adonis El Salloukh, Abdus Samad Ansari, Alexander Chiu, Divya Mathews

**Affiliations:** 1 Stanley Eye Unit, Abergele Hospital, Llanfair Road, Abergele, Conwy Wales, UK; 2 Section of Academic Ophthalmology, School of Life Course Sciences, FoLSM, King’s College London, London, UK

**Keywords:** Viscocanalostomy, Phaco viscocanalostomy, Uveitis, Uveitic glaucoma

## Abstract

**Background:**

Uveitic glaucoma commonly leads to a more intense optic nerve damage than other types of glaucoma, causing glaucomatous optic nerves and visual field defects. Anterior uveitis is the most commonly associated risk factor. Surgical intervention is usually indicated when all medical treatment has failed. We report five-year results for 16 eyes of uveitic glaucoma managed with viscocanalostomy (VC)/Phaco viscocanalostomy (PVC).

**Methods:**

Retrospective analysis on all uveitic glaucoma cases meeting a five-year follow up was completed. All patients were managed surgically with either viscocanalostomy (VC) or phacoviscocanalostomy (PVC). Outcomes evaluated included intraocular pressures measurement pre-listing, on day 1, year 1 to year 5. Complete success rate was defined as achieving an intraocular pressure (IOP) lower than 21 mmHg or reduced by 30% without medications, and qualified success was achieved when IOP was lower than 21 mmHg or a reduction in IOP of 30% with topical medical therapy ± Laser goniopuncture (LGP). If further surgeries were required to reduce IOP due to glaucoma progression then they were classified as a failure.

**Results:**

A total of 16 patients with uveitic glaucoma were reviewed. Complete success was seen in 75% of patients at year 1, 50% of patients at year 3 and 19% of patients in year 5. Conversely qualified success was achieved in 94% of patients at year 1, 86% of patients at year 3 and 75% of patients at year 5. In the group of patients requiring further surgery, 50% of patients had previous surgeries, including cataract surgery, trabeculectomy and viscocanalostomy. There was a mean number of 4 pre-operative drops before their primary surgery and a mean drop in eye medications of 1.1 at 5 years follow-up. Success rates were prognostically linked to lower mean number of interventions and lower percentage of previous surgeries.

**Conclusion:**

There remains a significant paucity of information in the utilization of PVC in uveitic glaucoma. The advantage of nonpenetrating glaucoma surgery (NPGS) includes the lack of entry into the anterior chamber and the avoidance of an iridectomy which may reduce intraocular inflammation and postoperative complications. Our study shows that non-penetrating surgery is successful in treating advanced uveitic glaucoma.

## Background

Uveitic glaucoma can commonly lead to a more extreme form of optic nerve damage in comparison to other types of glaucoma causing glaucomatous optic nerves and visual field defects [1]. Anterior uveitis is the most commonly associated risk factor [2, 3, 4]. However, the risk not only increases with the chronicity of active inflammation, [2, 3, 5, 6] but also the duration of corticosteroid use [2, 3] although the latter may reflect more severe inflammation.

The pathophysiological processes at play in uveitis are complex and multifactorial with cellular, biochemical and morphological changes occurring which can result in both fall and rise in IOP. Due to the complexity of the disease process, management requires a multidisciplinary approach with rheumatologists and glaucoma specialists to manage both the inflammation and glaucoma.

Surgical intervention is usually indicated when all medical treatment has failed. Studies indicate that about 30% of uveitic glaucoma patients lead to surgical intervention [7]. Postoperative increase in inflammation and scarring is a particular risk in uveitic patients. Traditionally trabeculectomy with MMC / 5FU may give the best outcome as the antimetabolite prevents primary failure due to scarring [7, 8]. However, trabeculectomy requires very frequent follow up for optimum results with assessment of the developing “bleb” and intraocular pressure with needling procedures and repeat antimetabolite when deemed appropriate. Authors have reported success with tube procedures [9, 10], however corneal decompensation and hypotony are an unfortunate recognized complication associated with this technique [9]. Both trabeculectomy and tube surgeries necessitate entering an eye which may have only precarious preoperative control of inflammation, with the risk of exacerbating the uveitic response.

For these reasons interest has risen in non-penetrating glaucoma surgery. Little has been published on outcomes although previous authors have postulated reported success [11]. Non-penetrating glaucoma surgery aims to avoid entering the anterior chamber and doing an iridectomy which effectively reduces the inflammation post-operatively [7]. In non-penetrating glaucoma surgery, bleb manipulation and post-operative antimetabolites injection are not required as it is bleb independent surgery. Deep sclerectomy and viscocanalostomy have been both reported to be successful in uveitic glaucoma in the literature with success rates ranging from 50 to 90% [12, 13, 14, 15].

We report the first five year outcome data for 16 eyes of uveitic glaucoma managed with viscocanalostomy (VC)/Phaco viscocanalostomy (PVC).

## Methods

We aimed to evaluate results for a five year follow up period in 16 patients diagnosed with uveitic glaucoma and managed surgically with either VC or PVC. The study followed the Tenets of the Declaration of Helsinki.

All patients underwent NPGS either VC or PVC at the Stanley eye unit in Abergele Hospital performed by the same surgeon, under subtenon’s anaesthesia (local anaesthesia).

Patients selected were all diagnosed with uveitic glaucoma and all patients were listed for surgery due to progression of glaucoma despite maximal medical therapy. Uncontrolled glaucoma was defined as progression of visual fields defects, progression of optic neuropathy determined morphologically on clinical examination and/or using optical coherence tomography, and IOP greater than 21 mmHg.

The inclusion criteria included: glaucoma secondary to uveitis, uveitic patients whose inflammation was as well controlled as possible before surgery, progressive optic neuropathy under maximum tolerated medical therapy. The exclusion criteria included: non uveitic glaucoma patients, peripheral anterior synechiae, and previous ocular surgery (excluding clear cornea cataract extraction and anti-glaucoma surgery).

Uveitis was defined based on the presence of anterior chamber cells and anterior chamber flares. The management was done according to the degree of anterior chamber cells and flare. All patients included in this study had mainly anterior uveitis. All patients included in this study had predominately anterior uveitis. All patients underwent blood tests including vasculitic screen and chest x-ray to look for a common systemic cause for recurrent chronic uveitis. The etiology of these uveitic patients included Fuch’s Heterochromic Cyclitis (FHC) and idiopathic uveitis. The diagnosis of FHC was made by the presence of the triad of chronic anterior uveitis, heterochromia and cataract. In patients were no specific cause of uveitis was identified the diagnosis of idiopathic uveitis was made. None of patients included were on immunomodulating therapy. No patient had posterior segment involvement. Glaucoma secondary to uveitis was defined as IOP greater than 21 mmHg, progressive optic neuropathy and visual defects that has been the result of uveitis. Uveitic glaucoma was managed surgically only when it was still progressing under maximal medical therapy.

No systemic medication was used prior to surgery to control the inflammation. Surgery was performed when the eye had been quiescent for at least 2 months. In case of FHC and in patients requiring urgent surgery in the presence of mild uveitis they were treated with topical Prednisolone 6 times a day for at least 1 week prior to surgery. All patients received subconjunctival dexamethasone at the end of the surgery. In the immediate post-operative period, patients had topical steroids 6 times a day (G. prednisolone acetate 1%), on a reducing regimen, and antibiotics 4 times a day (G. chloramphenicol 0.5%) for 4 weeks. All preoperative glaucoma medications were stopped after surgery.

Outcomes of interest were recorded including regular intraocular pressures measurement pre-listing, on day 1, year 1 to year 5. Additional outcomes evaluated included the need to restart glaucoma drops during follow-up. Goniopuncture neodymium-yttrium aluminum garnet (Nd-YAG) was performed if target IOP was not achieved by the surgical procedure, or if progression was noticed on visual fields. If an immediate drop in IOP was not achieved, the antiglaucoma drops were commenced.

## Results

A total of 16 patients with uveitic glaucoma were reviewed. Mean patient age was 68.31 ± 8.84 years. Out of the 16 patients, 10 patients were females and 8 patients were operated on their right eyes. 31.25% of patients had previous surgeries including cataract surgery (18.75%), trabeculectomy (6.25%) and viscocanalostomy (6.25%). All patients were followed up for 5 years under glaucoma specialist and medical retina specialist care. Primary care physicians referred 25% of the patients, with all other referrals being made by the optician or other units in North Wales. Table 1 shows the patient demographics, results expressed as percentage.

**Table 1:** Patient demographics for successful operations, those requiring repeated surgery and patients suffering from severe postoperative IOP. Results expressed as percentage

	Total population (N = 16)	Patients successfully operated on (n = 12)	Patients requiring repeat surgery (n = 4)	Patients with severe postoperative IOP (n = 11)
Mean age	68.31 (SD 8.84)	69.25	65.5 (SD 5.8)	70.36 (SD 9.29)
Percentage female	62.5	75	25	54.54
Percentage left eye operated	25	33.33	0	27.27
Patients with previous operations	31.25	25	50	45.45
Previous operation type				
None	68.75	75	50	54.54
Cataracts	18.75	16.67	25	27.27
Trab	6.25	0	25	9.09
Visco	6.25	8.33	0	9.09
Mean number of previous treatments	3.62 (SD (1.20)	3.5 (SD 1.31)	4 (SD 0.82)	3.73 (SD 1.10)
Percentage of patients with surgical type visco alone	68.75	16.67	75	45.45
Percentage of patients with perforation	12.5	8.33	25	9.09
Mean CDR	0.8 (SD 0.20)	0.78 (SD 0.16)	0.45 (SD 0.1)	0.65 (SD 0.23)

### Complete success vs qualified success

Complete success was considered if the post-operative IOP was reduced to below 21 mmHg or reduced by more than 30% compared to the pre-listing IOP without glaucoma drops (Table 2). Qualified success included those with or without the use of topical medication. Surgery was considered unsuccessful if further surgeries were required to reduce IOP due to glaucoma progression despite primary surgery and maximal medical therapy post-operatively.

**Table 2:** Complete vs Qualified Success for all study participants

	Year 1	Year 2	Year 3	Year 4	Year 5
Complete success	12/16 (75%)	11/16 (63%)	8/16 (50%)	5/16 (31%)	3/16 (19%)
Qualified success	15/16 (94%)	14/16 (86%)	14/16 (86%)	12/16 (75%)	12/16 (75%)

75% of the total population studied achieved a qualified successful outcome at year 5. In the group of patients requiring further surgery, 50% (n = 4) of patients had previous surgeries and had a mean number of 4 interventions prior to their first surgery. The group of patients who were successfully operated on had lower mean number of intervention and lower percentage of previous surgeries (Table 3). Table 3 showing outcome data on visual acuity, referrals made, percentage requiring drops and IOP.

**Table 3:** Outcome data at time points assessed, results for visual acuity, referrals made, percentage requiring drops and IOP

	Total population (N = 16)	Successful procedure (n = 12)	Patients requiring repeat surgery (n = 4)	Patients with severe postoperative IOP (n = 11)
Mean visual acuity				
Visual acuity day 1	− 13.43 (SD 11.06)	− 16.42 (SD 11.17)	− 5.21 (SD 5.71)	− 13.92 (SD 11.70)
Visual acuity year 1	− 12.24 (SD 10.92)	− 12.68 (SD 11.55)	− 10.24 (10.74)	− 15.78 (SD 12.02)
Visual acuity year 2	− 11.99 (SD 10.92)	− 10.77 (SD 9.14)	− 15.62 (SD 7.21)	− 14.26 (SD 10.19)
Visual acuity year 3	− 13.07 (SD 9.92)	− 11.12 (SD 11.77)	− 15.68 (SD 8.35)	− 18.62 (SD 9.01)
Visual acuity year 4	− 11.05 (SD 6.39)	− 13.82 (SD 3.91)	/	− 10.32 (SD 10.68)
Visual acuity year 5	− 11.54 (SD 13.46)	–	–	− 11.54 (SD 13.46)
Visual acuity year 6	− 11.81 (SD 13.47)	–		− 11.81 (SD 13.47)
Percentage of patients referred by GP				
	25	25	25	27.27
Percentage of patients requiring drops at follow-up				
6 months	18.75	8.33	50	27.27
9 months	12.5	0	50	18.18
12 months	43.75	25	100	63.63
18 months	43.75	33.33	75	63.63
24 months	50	41.67	75	72.72
30 months	50	41.67	75	72.72
36 months	56.25	50	75	81.81
42 months	50	41.67	75	63.63
48 months	37.5	33.33	50	45.45
54 months	43.75	41.67	50	54.54
60 months	37.5	33.33	50	45.45
66 months	37.5	33.33	50	45.45
72 months	12.5	8.33	25	18.18
78 months	0	0	0	–
84 months	6.25	8.33	0	9.09
Mean IOP				
Listing pressure	30.75 (SD 6.78)	29.50 (SD 7.08)	34.50 (SD 4.65)	33.45 (SD 5.98)
Day 1	13.43 (SD 8.53)	12.75 (SD 6.99)	15.5 (SD 13.30)	13.45 (SD 10.08)
1 week	16.25 (SD 9.97)	14.50 (SD 7.46)	21.50 (SD 15.64)	17.45 (SD 11.49)
1 month	20.25 (SD 7.97)	18.25 (SD 6.20)	26.25 (SD 10.66)	21.91 (SD 8.84)
3 months	16.73 (SD 5.32)	15.41 (SD 4.94)	22.00 (SD 3.46)	16.90 (SD 6.34)
6 months	19.13 (SD 11.05)	15.33 (SD 5.74)	34.33 (SD 15.50)	21.30 (SD 13.16)
9 months	18.85 (SD 10.07)	15.7 (SD 4.02)	29.33 (SD 17.93)	20.40 (SD 11.88)
12 months	17.07 (SD 4.56)	16.0 (SD 4.50)	17.00 (SD 2.64)	17.90 (SD 4.74)
24 months	15.86 (SD 4.20)	15.54 (SD 4.59)	12.33 (SD 3.51)	16.70 (SD 4.62)
30 months	15.64 (SD 3.59)	16.54 (SD 3.17)	21.00 (SD 7.81)	15.50 (SD 4.06)
36 months	15.85 (SD 4.69)	14.45 (SD 2.50)	14.50 (SD 2.12)	16.20 (SD 5.37)
42 months	16.40 (SD 3.27)	16.88 (SD 3.44)	18.50 (SD 0.71)	16.33 (SD 4.13)
48 months	16.00 (SD 2.97)	15.44 (SD 3.00)	12.00 (SD 2.82)	16.14 (SD 3.72)
54 months	15.40 (SD 2.91)	16.25 (SD 2.37)	16.50 (SD 6.36)	14.50 (SD 3.33)
60 months	15.70 (SD 4.00)	15.50 (SD 3.82)	–	14.33 (SD 4.67)
66 months	15.00 (SD 3.16)	15.00 (SD 3.16)	–	15.50 (SD 2.12)

Mean visual acuity was stable in the overall population. In fact, pre-operatively, mean visual field defect was -13.43 ± 11.06 and remained stable post operatively in year 5 (-11.54 ± 13.46) and year 6 (-11.81 ± 13.47). Mean IOP in the overall population pre-listing was 30.75 ± 6.78 mmHg. It dropped on day 1 to 13.43 ± 8.53 mmHg and by year 1 it was 44.49% lower than the pre-listing IOP. In the successfully operated group, mean IOP was dropped by 45.76% by 12 months and 46.13% by 60 months and shows that viscocanalostomy alone produces excellent long-term results. Interestingly, only a third of this group required drops after 5 years follow-up. In the group where further surgery was required, 50% of the patients needed drops at 54 months but also showed a drop in IOP by 52.17% from the pre-listing IOP.

### Additional analysis

#### 1. Comparison of pre and post op IOP using paired T-test (listing versus day 1)

##### Overall summary

The surgical procedure was successful in reducing IOP, whereby a statistically significant reduction (t = 6.001, p < 0.0001) in the IOP was observed when comparing the listing (mean listing pressure: 30.75 SE 1.69) and day 1 postoperative pressure (mean postoperative pressure: 13.43 SE 2.13).

#### 2. ANOVA comparison of change in IOP over time

##### Overall summary

One-way analysis of variance (ANOVA) was performed to ascertain whether intraocular pressure was reduced in the post-operative period, as assessed across fourteen time points over 5 years. Time points evaluated in this analysis include the preoperative, day one, week one, month one, month three, and for subsequent bi-annual follow-ups for duration of 5 years. We were unable to include month eighteen in this analysis due to the large number of patients failing to attend these assessment dates. A statistically significant change in intraocular pressure was observed between time points, (F = 5.01, p < 0.00001). A Tukey post-hoc analysis revealed findings observed in the one-way ANOVA are largely driven by the difference in mean IOP observed between preoperative and day 1 measurements (mean difference 17.31, HSD test 9.16, p < 0.05). The Tukey-post-hoc analysis also demonstrates all time points assessed against the preoperative IOP showed statistically significant reduction.

#### 3. Survival curves

**Fig. 1:**
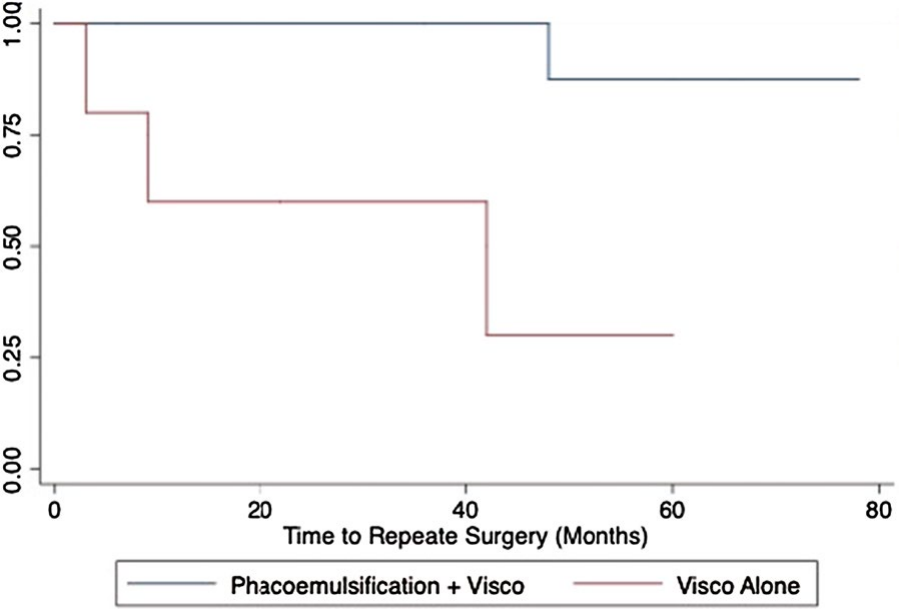
Kaplan–Meier assessing impact of type of surgery (VC vs PVC) of time to repeated surgery. (Log Rank: chi 6.45, p = 0.01)

**Fig. 2:**
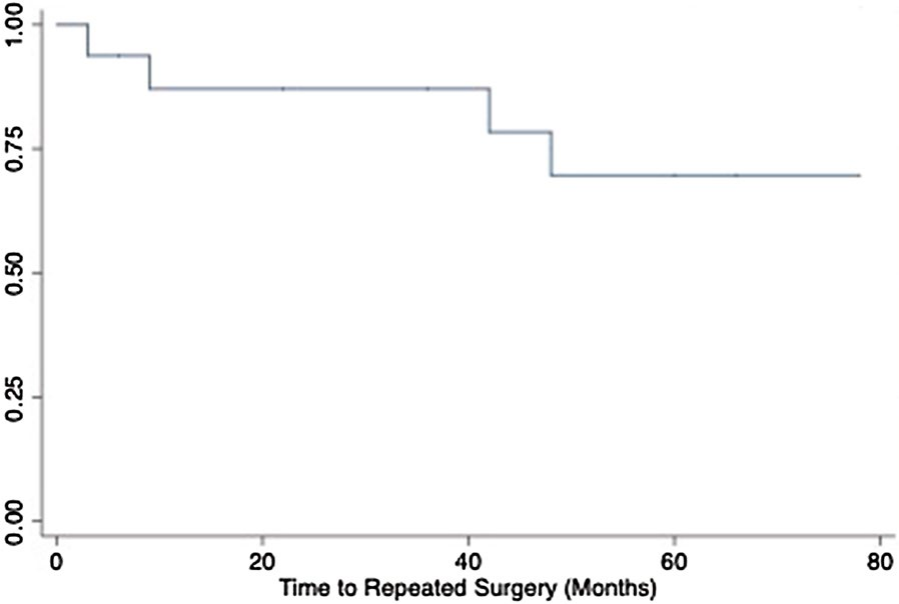
Kaplan–Meier survival estimate of time to repeated surgery

For the purpose of this project we have evaluated patients over a five year time frame. The outcome of this analysis is time until repeated surgery. Repeated surgery was confirmed via retrospective case review, where the requirement for repeated surgery would be documented carefully in the patient notes. Only those with confirmatory surgical notes were included in the study. (Figs. 1, 2).

#### 4. Cox proportional hazards model

##### Summary of cox proportional hazards model

Cox Proportional Hazard (PH) model expresses the hazard for patient drop out at a specific time for an individual given the status of their set of explanatory variables Hazard Ratio: 11.62 SE 13.78, p = 0.039. The variables collected from this cohort of patients have been described above. After establishing the significant association between type of surgery and risk for early repeated surgery; we wished to quantify the magnitude of effect. Results from the Cox PH main effects model suggests that the type of surgery patients underwent is important for predicting the variance in survival time; these results suggest that 1) participants receiving operations employing the isolated VC procedure are at 11.62 times higher risk of undergoing repeated surgery. We then assessed the PH assumptions for this final model with interactions using the KM and log–log survival curve visual assessments as well as the Schoenfeld residuals test.

##### Summary of Kaplan Meier Curves

Graphical evaluation of the basic Kaplan Meier curves (unadjusted) for each variable or the log–log survival curves (adjusted for each variable) allows us to visually determine whether the curves cross over, thus violating the PH assumption (Fig. 1). Provided the hazards cross for two or more categories of predictors, the PH assumption is not maintained. During variable selection we chose to assess the KM curves (unadjusted) for all categorical and binary variables in this dataset. Log-rank tests are also provided beneath the curves. Our only categorical variable included in our final model was surgical technique—PVC, versus VC alone. This variable met the visual assessment for the proportional hazard assumption, where the KM curve does not visually cross over (Fig. 2).

There were no significant surgical complications in the overall population studied. Elevated postoperative IOP was defined as post-operative IOP above 30 mmHg. 11 out of 16 patients experienced elevated IOP that eventually settled with medical management. Trabeculo Descemet’s window perforation was encountered in only two patients (12.5%) and had no long-term repercussions. None of the eyes had endophthalmitis, flat anterior chamber, surgically induced cataract or a flare-up of their uveitis. Four patients in total needed in goniopuncture in subsequent follow-ups post-operatively.

## Discussion

Managing concurrent uncontrolled uveitic glaucoma represents a surgical challenge. The surgical success rates in uveitic glaucoma varies markedly (50%–100%) [3]. There is a consensus that the surgical success rate of filtering surgery is lower for eyes with UG compared with primary open-angle glaucoma (POAG) [6].

Trabeculectomy is hailed as the surgical procedure of choice in uveitic glaucoma [12]. Although use of antiproliferative agents is controversial, some studies suggest a benefit in terms of long-term IOP control [12]. Stavrou et al. quote a 5-year success rate (IOP < 21 mmHg) as 78% in their uveitic glaucoma patients following trabeculectomy without antimetabolites, and with topical medication [9]. With the use of antimetabolites (5 FU or MMC), Ceballos et al. reports a cumulative probability of complete or qualified success as 78% at 1 year and 62% at 2 years in uveitic glaucoma patients [10]. However, in this group, 51.6% needed cataract surgery and 25% required repeat trabeculectomy. A retrospective study by Iwao et al. concluded that trabeculectomy with mitomycin c (MMC) in UG eyes is independently associated with a worse prognosis than the same procedure in POAG eyes after adjusting for all confounding factors [11].

Trabeculectomy with or without antimetabolites has well-documented complications such as hypotony maculopathy, aqueous leak, postoperative hyphema, worsening of intraocular inflammation, serous choroidal detachment, shallow or flat anterior chamber and bleb encapsulation [10, 11]. Although the use of antimetabolites can improve the success rate of glaucoma surgery, it also increases the severity of complications [16].

In cases with high risk of trabeculectomy failure such as aphakic patients, juvenile idiopathic arthritis, previous trabeculectomy failure, retinal detachment surgery, active inflammation the surgical procedure of choice is a glaucoma drainage device (GDD). Studies have shown that all 3 common types of GDD (Ahmed, Baerveldt, and Molteno) are effective in lowering intraocular pressure and reducing the number of glaucoma medications [17–20]. The most common complications after GDD in patients with uveitic glaucoma are: encapsulated bleb, transient hypotony, and hyphema, occlusion of the tube by inflammatory materials and corneal decompensation [6].

In an attempt to lower the incidence of all these complications following trabeculectomy or GDD, NPGS was developed.

The advantage of nonpenetrating procedures, such as deep sclerectomy (DS) and VC, is that there is no entry into the anterior chamber and the avoidance of an iridectomy may reduce intraocular inflammation and postoperative complications, which makes them of specific interest in uveitic glaucoma. In particular, VC, by attempting to restore an aqueous outflow pathway, is predominantly independent of external filtration and may improve clinical outcomes even in the presence of chronic inflammation [7]. Furthermore, in NPGS, bleb manipulation is not required as it is bleb independent and subsequent anti-metabolite sub conjunctival injections are not needed. The conjunctiva has little or no scarring leaving room for future glaucoma surgeries if needed. However, one must be aware that this procedure is likely to be unsuccessful in UG patients with peripheral anterior synechiae (PAS) as the outcome depends on a clear trabecular Descemet’s membrane window (TDW).

Souissi et al. [13] reported a mean decrease of IOP by 52.9% in eight eyes that underwent DS without antimetabolites with mean follow-up of 42.2 months. Complete success was obtained in 50%, relative success was obtained in 37.5% and failure in 12.5%. Additionally, Dupas et al. [14] found a success rate of 89% at 12 months in the group of patients that underwent trabeculectomy with MMC and a success rate of 88% in the group that underwent DS with MMC and with implant but with an increase number of postoperative adjustments in the DS group such as goniopuncture in 45% of the cases and needling in 15%. Similar results have been reported by Obeidan et al. [12] in patients that underwent DS with implant where a complete success rate was achieved in 84.6%, a qualified success was achieved in 7.7% and a complete failure in 7.7%.

Miscrocchi et al. [7] reported a qualified success in 90.9% with a mean IOP reduction of 46.4% in all eyes that had VC and a mean final IOP of 18.1 mmHg. In our study, qualified success was achieved in 94% of patients at year 1, and 75% of patients at year 5 with a mean final IOP of 15.70 ± 4.00 mmHg. In the group of patients requiring further surgery, 50% of patients had previous surgeries. There was a mean drop in eye drops at 6 months of 0.25, at 1 year of 0.5 mean and at 5 years of 1.1 post-operatively.

The only complication encountered was TDW perforation which occurred in 2 patients. None of the eyes had endophthalmitis, flat anterior chamber, surgically induced cataract or a flare-up of their uveitis.

The overall outcome of VC in this study is very encouraging yielding an overall success rate of 9/10 (90%). The average reduction in IOP in this study was 49.6%.

To date there are no studies on PVC in uveitic glaucoma. Combined phaco-trabeculectomy has been reported to be less successful than trabeculectomy alone [21, 22] and most glaucoma surgeons advise the use of MMC in combined procedures [23]. It has been suggested that the reason for a worse outcome following combined procedure is the added inflammation occurring from the cataract extraction giving rise to an increase in release of inflammatory mediators such as transforming growth factor-beta 1, which promote subconjunctival/episcleral scarring and failure of the filtering bleb [21, 22]. By performing PVC, these aqueous derived vasoactive stimulators of fibroblast activation cross the TDW into Schlemm’s canal and probably leave the eye either through existing collector channels, or through the uveoscleral outflow and therefore do not come into contact with the episcleral/subconjunctival space fibroblasts as they would after trabeculectomy [24].

There are several limiting factors in this study. This is a retrospective case series with limited sample size and the included eyes were heterogeneous based on type of uveitis, type of procedure and lens status.

None of our patients were on immune-modulating medication and this may have affected our success rate. Also, of relevance is that we only had idiopathic uveitis and Fuch’s heterechromic cyclitis (FHC) and hence a direct comparison with other studies (with different uveitic aetiology) is not possible. The limited sample size, heterogeneous subjects further reduces the possibility for analytical approach to the data. Also relevant is that the results are of surgery in a subset of patient with mild form of uveitis and cannot be easily generalized to other types of uveitis.

## Conclusion

To our knowledge, our study is the second report on viscocanalostomy in uveitic glaucoma (UG). It has shown that viscocanolostomy should be considered as a surgical option in the management of uveitic glaucoma. It provides a potential further surgical alternative in uveitic glaucoma that are difficult to control with topical treatment.

## Data Availability

The datasets generated and/or analysed during the current study are not publicly available as they include confidential patient information, but anonymised forms of the data are available from the corresponding author on reasonable request. Patient relevant data and materials are all held by Betsi Cadwaladr University Health Board.

## Abbreviations

PVC: Viscocanalostomy (VC)/Phaco viscocanalostomy
LGP: Laser goniopinture
NPGS: Nonpenetrating glaucoma surgery
DS: Deep sclerectomy
IOP: Intraocular pressure
Nd-YAG: Goniopuncture neodymium-yttrium aluminum garnet
ANOVA: One-way analysis of variance
PH: Cox Proportional Hazard
KM: Kaplan Meier
POAG: Primary open-angle glaucoma
GDD: Glaucoma drainage device
PAS: Peripheral anterior synechiae
MMC: Mitomycin c
UG: Uveitic glaucoma
FHC: Fuch’s heterechromic cyclitis
TDW: Trabecular descemet window
